# Clinicopathological analysis of head and neck rhabdomyosarcoma: A series of 10 cases and literature review

**DOI:** 10.4317/medoral.22106

**Published:** 2018-02-25

**Authors:** Flávia-Sirotheau-Correa Pontes, Jonas-Ikikame de Oliveira, Lucas-Lacerda de Souza, Oslei-Paes de Almeida, Eduardo-Rodrigues Fregnani, Rafael-Sarlo Vilela, Wanessa-Miranda Silva, Felipe-Paiva Fonseca, Hélder-Antônio-Rebelo Pontes

**Affiliations:** 1 Service of Oral Pathology, João de Barros Barreto University Hospital, Federal University of Pará (Belém/Brazil); 2 Department of Oral Diagnosis (Pathology), Piracicaba Dental School, University of Campinas (Piracicaba/Brazil); 3 Department of Oral Medicine, Sírio-Libanês Hospital, São Paulo/Brazil; 4 Department of Pathology, Sírio-Libanês Hospital, São Paulo/Brazil; 5 Department of Oral Surgery and Pathology, School of Dentistry, Universidade Federal de Minas Gerais (Belo Horizonte/Brazil)

## Abstract

**Background:**

To describe the clinicopathological characteristics of a series of head and neck rhabdomyosarcoma (RMS) and to review the literature.

**Material and Methods:**

Cases diagnosed as RMS affecting the head and neck region were retrospectively retrieved from the files of two Brazilian institutions from January 2006 to January 2017. Data on clinical features (sex, age and affected site), microscopic subtype, immunohistochemical results, treatment employed and follow-up status were obtained from the patient’s medical charts.

**Results:**

During the period considered, 10 cases of RMS were identified. Females predominated (4M:6F), the mean age at diagnosis was 16.5 years-old and the orbit was the most affected site (4 cases). Microscopically, most cases were classified as embryonal RMS (6 cases) and the Desmin/Myogenin/Myo-D1 immunohistochemical positivity was useful to confirm the diagnosis. Chemotherapy and radiotherapy were applied to 9 and 8 patients respectively, whereas 2 patients were treated by surgery. Recurrences occurred in 3 patients and distant metastasis in 2 cases. Nine patients were alive in their last follow-up, 3 of them with disease, whereas 1 patient died due to the disease.

**Conclusions:**

Head and neck RMS is an aggressive malignant neoplasm which demands especial concern to achieve early diagnosis and successful treatment.

** Key words:**Rhabdomyosarcoma, soft tissue tumors, head and neck, oral cavity, chemotherapy.

## Introduction

Rhabdomyosarcoma (RMS) is classified by the World Health Organization as a skeletal muscle tumor arising from undifferentiated skeletal tissue (1,2), predominantly affecting the head and neck region, with approximately 40% of the cases involving this area (3-5). RMS is the most common soft tissue sarcoma in children, accounting for 4.5% of all pediatric malignant neoplasms and approximately 50% of the solid malignancies diagnosed in patients under 10 years old (6). On the other hand, adult RMS is more commonly observed in the extremities, rarely affecting the head and neck (7).

RMS are highly sensitive to chemotherapy and radiotherapy, as a consequence, over the last 30 years pediatric patients had a significant improvement in their prognosis, with the 5-year survival rates achieving 80% to 85% in some series (4,8,9). Nevertheless, the outcome for adults is not as satisfactory as for the pediatric patients and both children and adults are currently treated by aggressive surgical resections followed by chemotherapy and radiotherapy (10).

In this study we aim to describe the clinicopathological characteristics of a series of head and neck RMS.

## Material and Methods

All cases diagnosed as RMS affecting the head and neck region were retrospectively retrieved from the files of the Oral Pathology Service of the João de Barros Barreto University Hospital (Belém/Brazil) and from the Pathology Department of the Sírio-Libanês Hospital (São Paulo/Brazil) from January 2006 to January 2017. Data on clinical features (sex, age and affected site), microscopic subtype, immunohistochemical results, treatment employed and follow-up status were obtained from the patient’s medical charts and descriptively presented. This study was approved by the local Ethical Committee.

## Results

**Figure 1:**
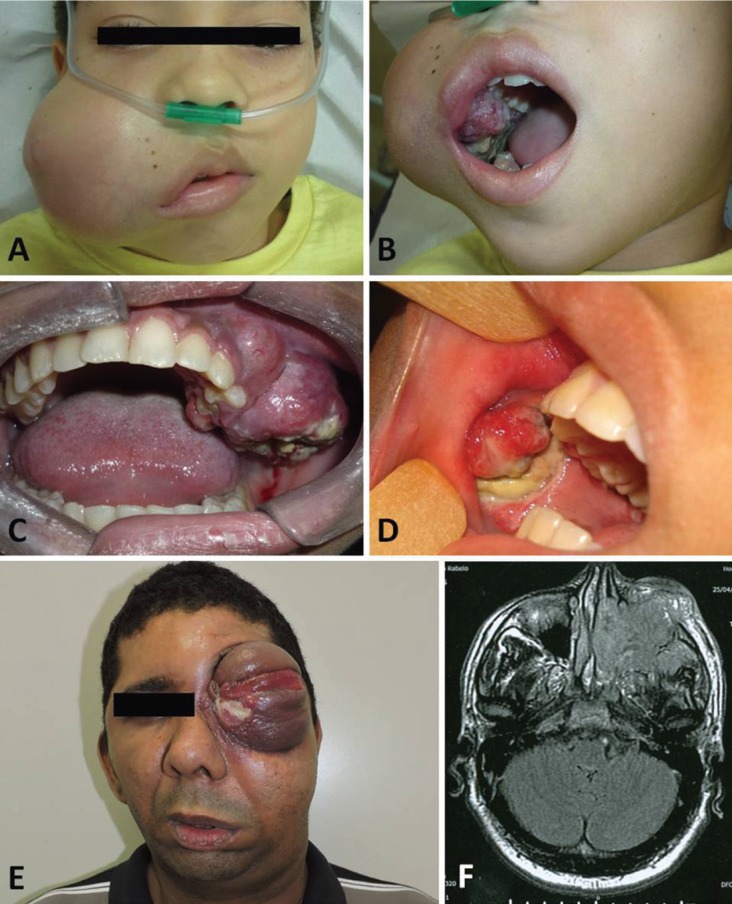
Clinical presentation of the three cases of RMS affecting the oral cavity. A) Case 1. Embryonal RMS causing a significant facial asymmetry. B) Intra-oral exam revealed a diffuse ill-defined soft-tissue neoplasm with irregular surface and areas of necrosis. C) Case 2. Embryonal RMS affecting the maxillary mucosa of a 23 year-old female patient causing a painful swelling with ulcerative regions. D) Case 3. Embryonal RHS affecting the cheek mucosa of a 10-year old female patient. Areas of necrosis are easily seen. E) Clinical presentation of one case of RMS affecting the orbit and the maxillary sinus of a 38-year-old male patient. F) Computed tomography demonstrated that left maxillary sinus was completely obliterated by the neoplasm.

**Figure 2:**
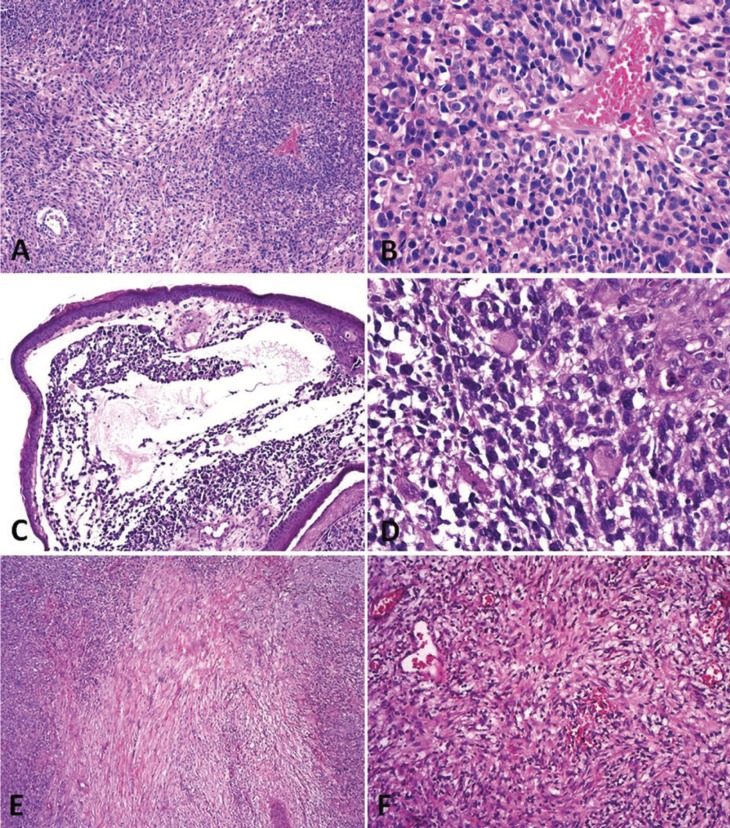
Microscopic findings of RMS. A and B) Case 1 diagnosed as embryonal RMS consisting of undifferentiated neoplastic cells, with atypical mitosis and extensive areas of necrosis (H&E; 100X and 200X, respectively). C and D) Case 2 diagnosed as embryonal RMS with poorly differentiated small round cells, and scattered rhabdomyoblasts (H&E; 100X and 200X, respectively). E and F) Case 3 classified as spindle cell RMS predominantly composed of elongated malignant cells (H&E; 100X and 200X, respectively).

During the 11-year period investigated, 10 cases diagnosed as RMS were identified. The clinical and pathological data of these patients are summarized in  Table 1. Briefly, there was a slight female predominance (4M:6F;) with a mean age at diagnosis of 16.5 years-old (range 6 to 38 years). The orbit was the most affected site (4 cases), followed by the oral cavity (3 cases) (Fig. 1). Microscopically, most of the cases presented as embryonal RMS (6 cases) characterized by small, round, hypercromatic neoplastic cells with the so-called rhabdoid cells showing large eosinophilic cytoplasm and displaced nuclei. Two cases presented as undifferentiated high-grade sarcomas with severe cellular pleomorphism, frequent atypical mitoses and variable areas of necrosis. One case was classified as the spindle cell variant characterized by elongated neoplastic cells with scarce eosinophilic cytoplasm and the presence of “herringbone” growth pattern in some areas, and one case of the alveolar variant with neoplastic nests presenting loosely arranged central cells and peripheral ones tightly attached to the surrounding connective tissue (Fig. 2).

**Table 1:**
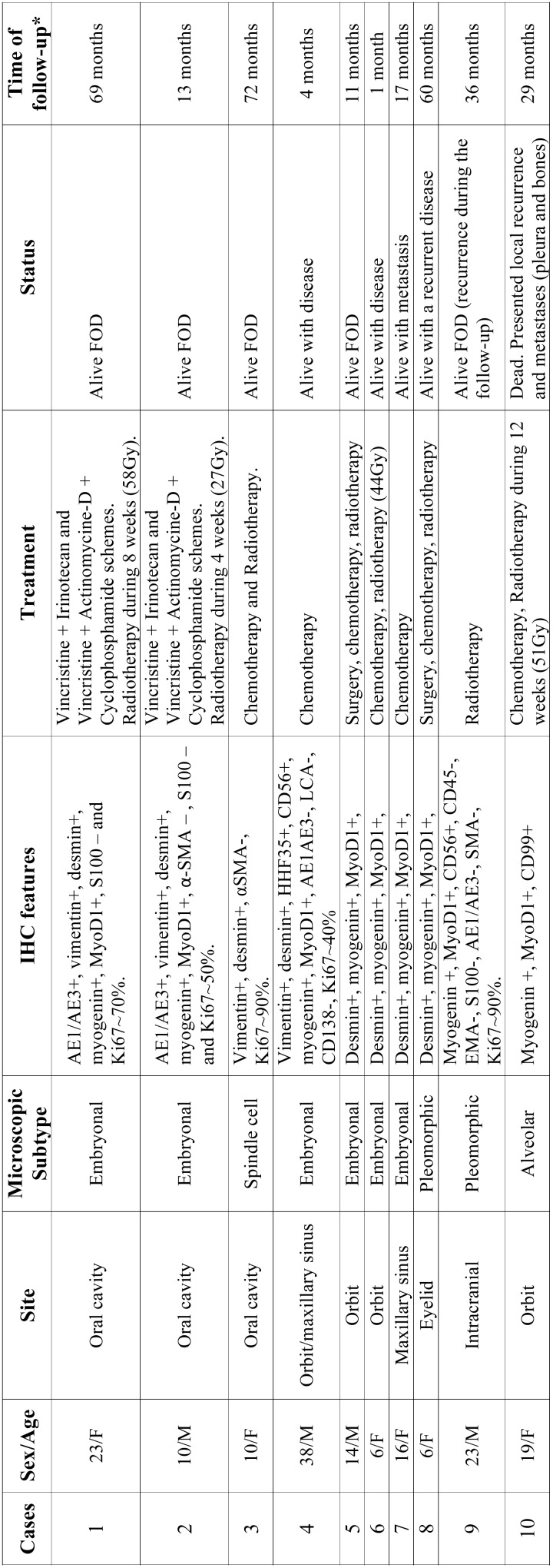
Clinicopathological and follow-up data of 10 cases of head and neck rhabdomyosarcoma. * Time of follow-up comprises the time difference between the diagnosis and the date of last follow-up or the date of death.

**Figure 3:**
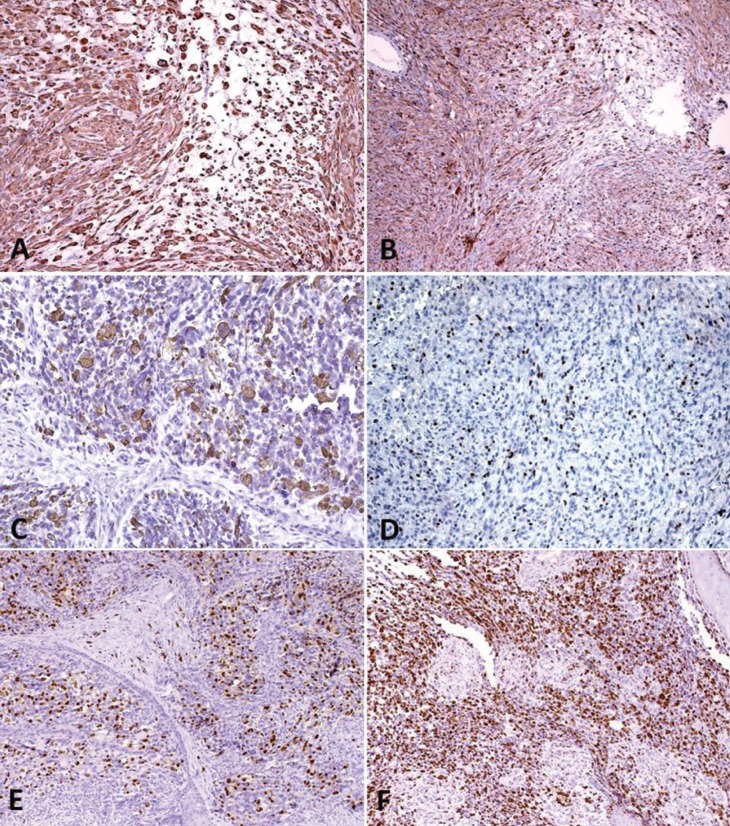
Immunohistochemical features of RMS. A) Vimentin (DAB; 200X), B) Cytokeratin (DAB; 100X), C) Desmin (DAB; 200X), D) MyoD1 (DAB; 100X) and E) Myogenin (DAB; 100X). F) Ki67 proli-ferative index achieved 90% in some cases (DAB; 100X).

All cases were positive for desmin, myogenin and MyoD1 antigens. Pan-cytokeratin (AE1/AE3) was expressed in two cases and Ki67 proliferative index was high in all tumors ranging from approximately 70 to over 90% (Fig. 3). Nine patients received chemotherapy, and radiotherapy was applied to 8 individuals. Only 2 patients were submitted to surgery. Nine patients were alive after a follow-up period that ranged from 1 to 72 months. However, recurrences were seen in 3 cases and distant metastases in 2 cases. Three patients remained alive with disease, and 1 patient died of disease.

## Discussion

RMS is a mesenchymal malignant neoplasm with skeletal muscle differentiation that represents the most common soft tissue sarcoma in the pediatric population. Approximately 40% of the cases develop in the head and neck region and the appropriate treatment demands a multi-modality approach (5,6,11-13). In this study we described a series of 10 cases of head and neck RMS and reviewed all published clinical series dealing with RMS of the head and neck with at least 3 cases reported to better understand the clinicopathological features of this aggressive malignancy (Table  Table 2, Table 2 continue,  Table 2 continue-1,  Table 2 continue-2,  Table 2 continue-3.).

**Table 2:**
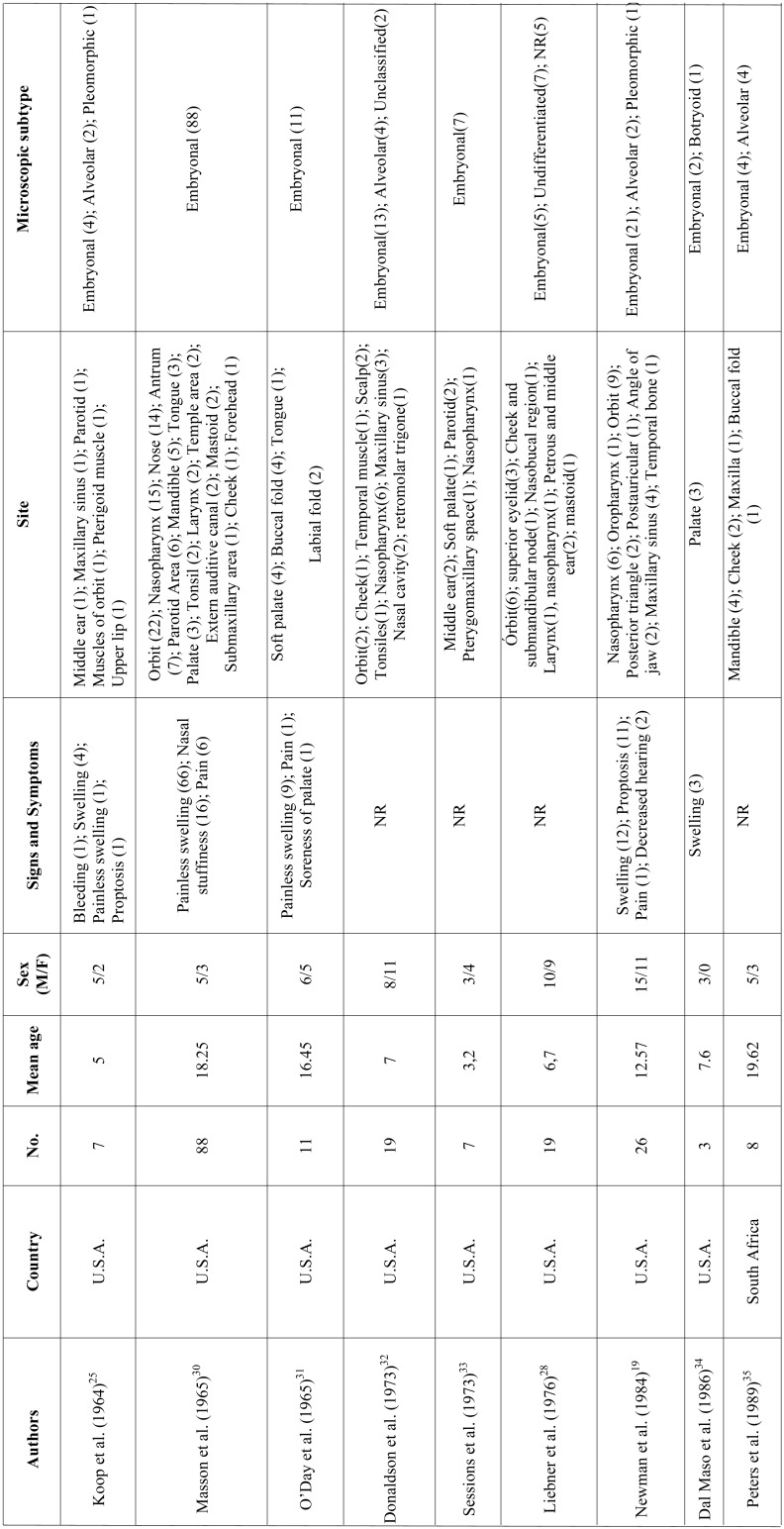
Clinicopathological features of head and neck RMS presented in the series (with at least 3 cases) previously published in literature.

**Table 2 continue:**
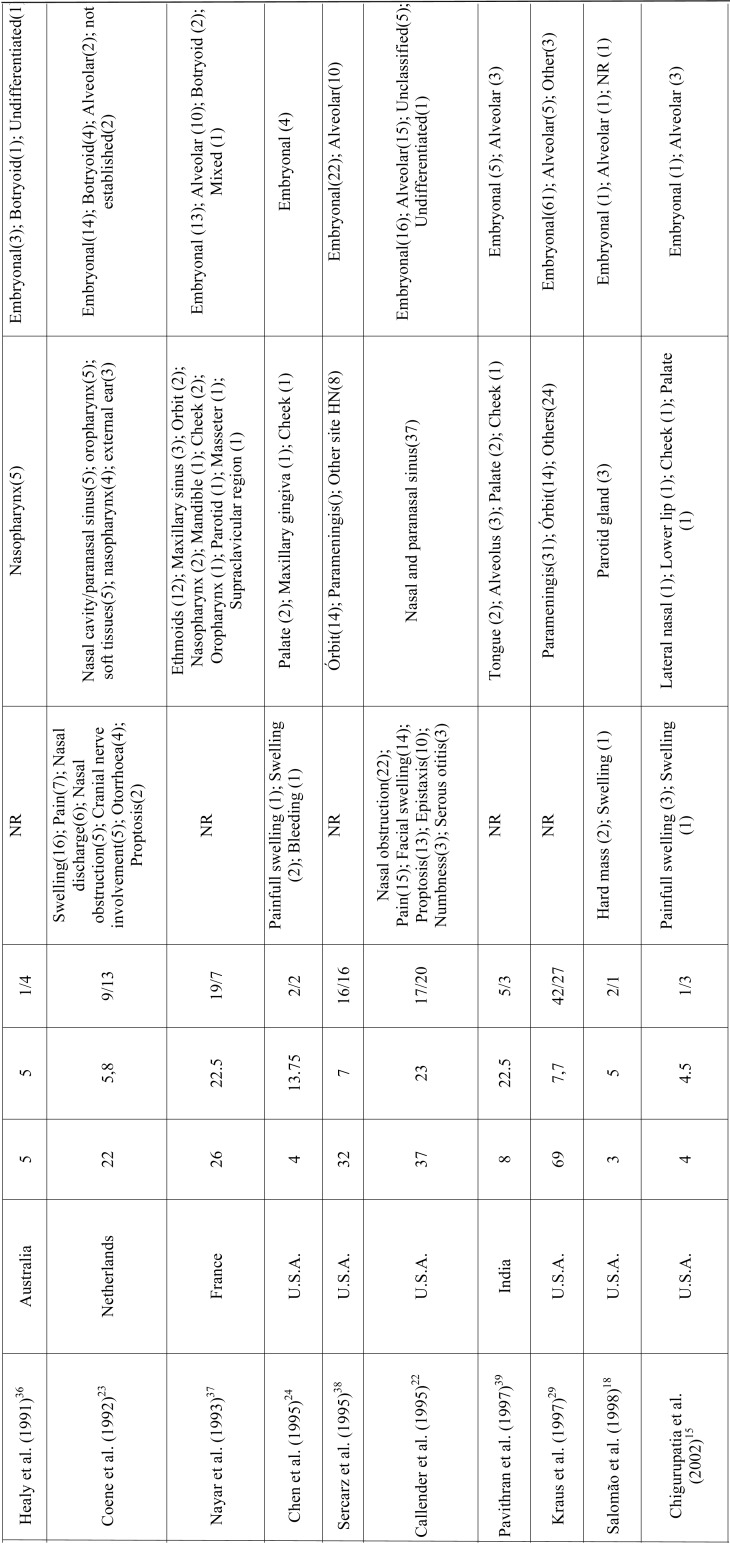
Clinicopathological features of head and neck RMS presented in the series (with at least 3 cases) previously published in literature.

**Table 2 continue-1:**
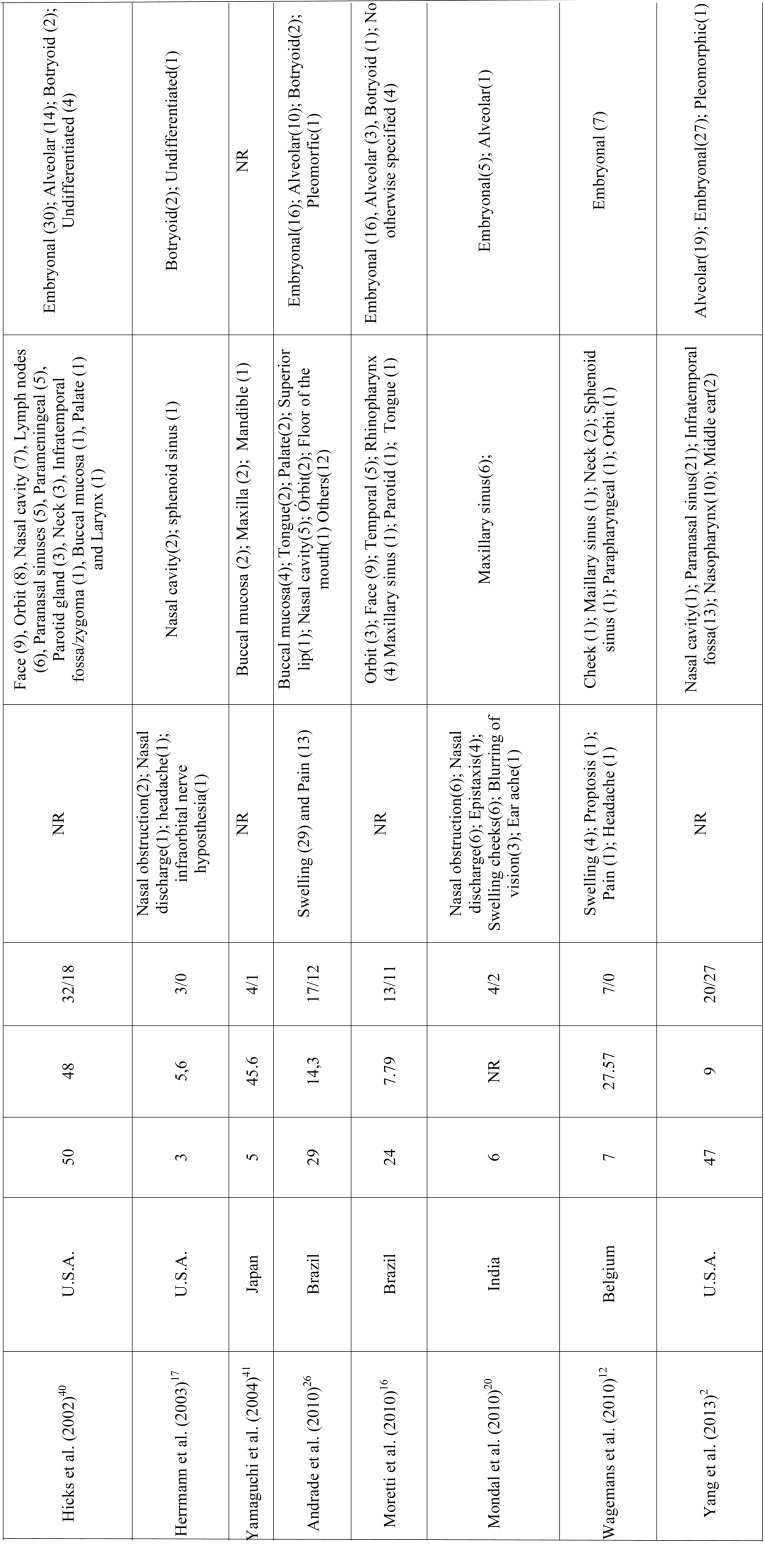
Clinicopathological features of head and neck RMS presented in the series (with at least 3 cases) previously published in literature.

**Table 2 continue-2:**
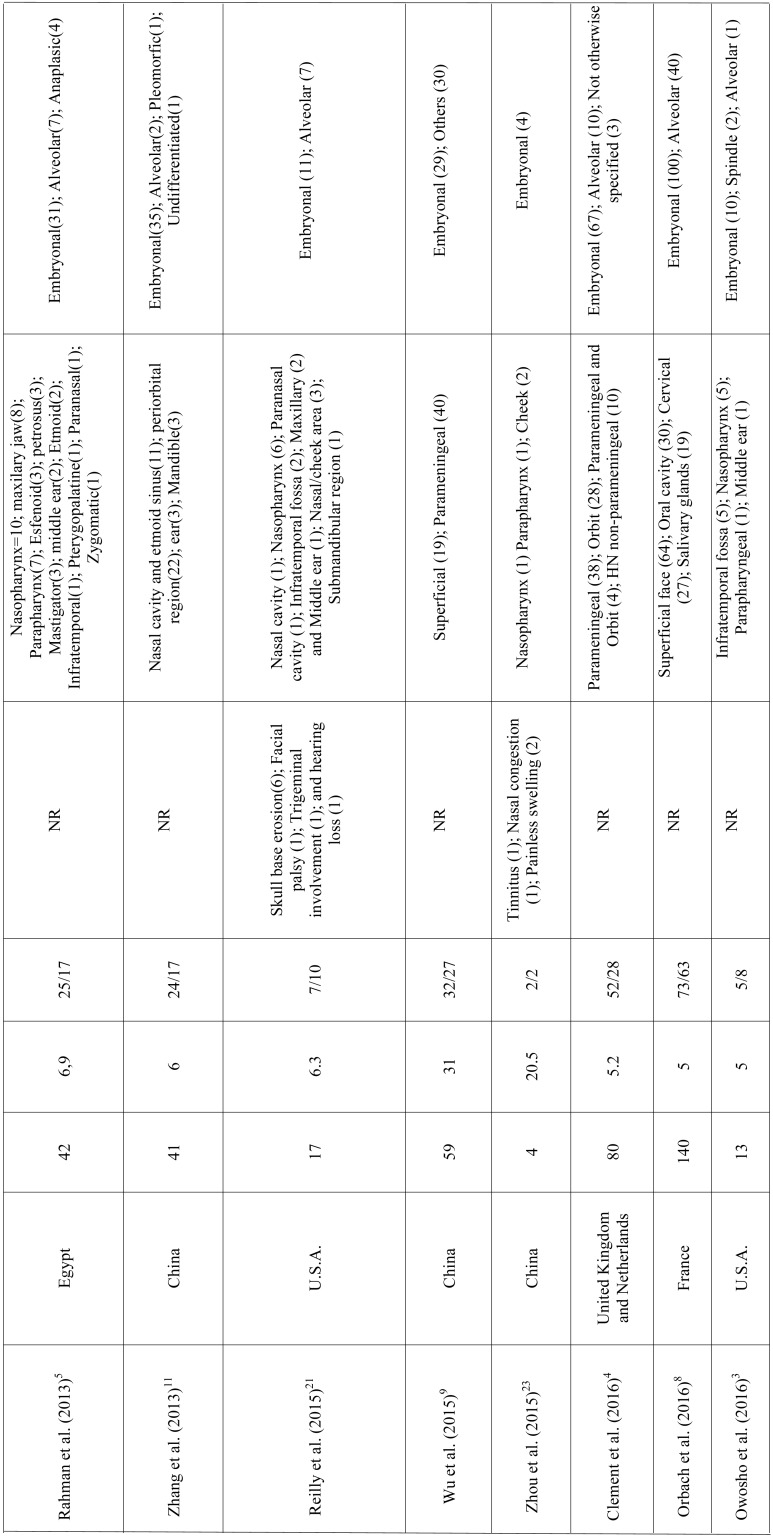
Clinicopathological features of head and neck RMS presented in the series (with at least 3 cases) previously published in literature.

**Table 2 continue-3:**
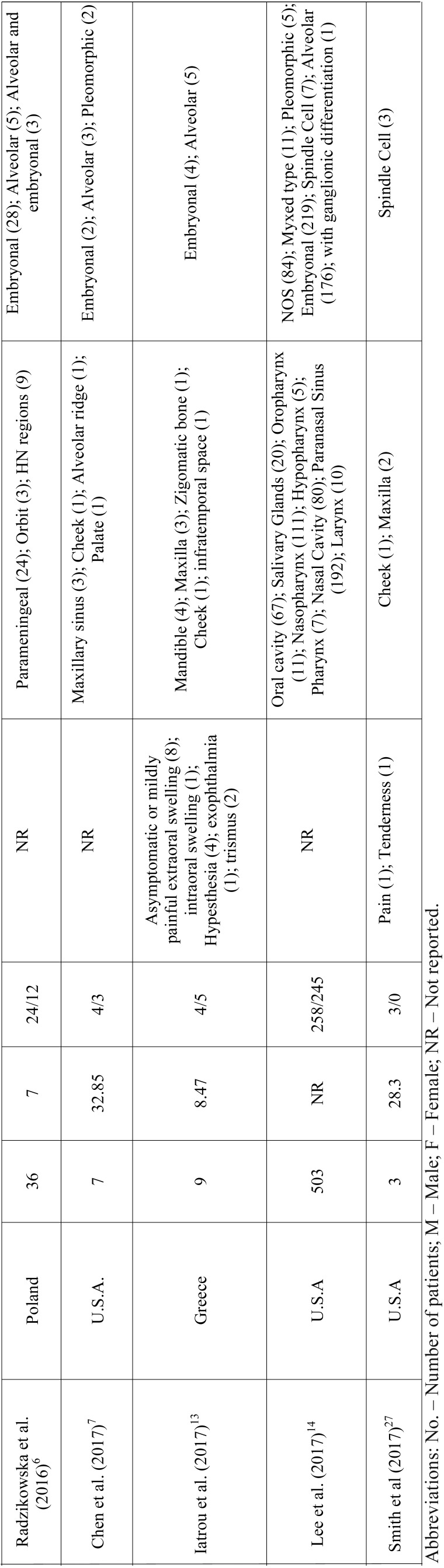
Clinicopathological features of head and neck RMS presented in the series (with at least 3 cases) previously published in literature.

In contrast to our series where females predominated, in the literature RMS presents a slight male predilection, with a male:female ratio of 1.5:1 (5,6,11,12,14). Clinical signs and symptoms mainly depend on the affected site and may vary considerably. Some cases are asymptomatic, although a painful swelling is the most common clinical manifestation in the head and neck region, usually causing facial asymmetries(15-19). Other complains may also be observed, including proptosis, nasal stuffiness and nasal discharge(15,19-24). In addition, as observed in one case of our series where an infectious lesion was initially clinically considered, misdiagnosis may also occur, potentially leading to an incorrect therapeutic approach and significant delay to achieve the correct diagnosis (20,25).

Microscopically, RMS can be classified into different histologic subtypes, and as shown in our study, the most common is the embryonal subtype (EMB), accounting for 60% of all cases, characterized by undifferentiated, small, round and hypercromatic cells with variable number of strap or tadpole-shaped, eosinophilic rhabdomyoblasts (11,20). Alveolar subtype (ALV) represents approximately 30% of the cases, and it is characterized by small round rhabdomyoblasts arranged in nests separated by connective tissue trabeculae and focal areas of alveolar architecture with hypercromatic nuclei and eosinophilic cytoplasm (11,15,16). ALV RMS is more common in older patients than EMB, ranging between 10 and 25 years-old with no gender predilection and usually with a more unfavorable prognosis (8,11,16,26). Moreover, approximately 75% of ALV carry a chromosomal translocation that results in the fusion of two transcript factor-encoding genes, the PAX3 gene (or less commonly PAX7 gene) and the FOXO1 gene, resulting in the expression of the chimeric PAX3/7-FOXO1 protein (7).

Other less common variants include the pleomorphic RMS that only rarely occurs in the pediatric group and comprises about 5% of all cases diagnosed (11,17); the spindle cell subtype that has previously been considered a variant of the EMB, but it is now recognized as a separate subtype (1); and the botryoid variant that represents an EMB subtype with a grapelike macroscopic and histologic appearance caused by sub-epithelial tumor aggregates (21). More recently, a sclerosing RMS was also recognized (27). In our series, two cases were diagnosed as pleomorphic RMS, one case showed features consistent with the spindle cell variant and one was classified as ALV. Immunohistochemistry is very important to confirm the diagnosis, especially in undifferentiated cases, and to exclude other neoplasms with cells demonstrating rhabdomyoblast-like features. Positivity to desmin, myogenin and MyoD1, as demonstrated in this study, is the main profile currently used.

Significant improvements were achieved in the treatment of RMS over the last decades and multimodality treatment has been established as the recommended therapy for these patients with a combination of chemotherapy, radiation, and surgery. In cases where anatomical location allows total tumor resection, surgery is indicated followed by radiotherapy and chemotherapy. Where free surgical margins are not possible to be obtained, chemotherapy and radiotherapy is applied(6,18). In our series, most cases were treated by chemotherapy combined with radiotherapy, whereas only two patients were submitted to surgical resection of their tumors; this finding is explained by the advanced tumor stages observed, some of them very close to vital structures, which impaired an adequate removal.

The most common cause of death is tumor progression and involvement of adjacent structures (8,10,12,19). Regarding distant metastases, the most commonly involved site is the lung (5,7,28,29), but other locations can also be affected (28). In our sample, three patients presented local recurrences, and two distant metastases.

Primary location of the disease may significantly influence the patients’ outcome, since parameningeal areas, paranasal sinus, nasal cavity, mastoid area and infratemporal fossa tends to present a poorer prognosis than non-parameningeal cases, which may be consequence of the impossibility to achieve total resection of the neoplasms and due to their proximity to intracranial area (7,10). The size of the tumor in the moment of the diagnosis may also represent an important factor, with lesions greater than 5cm presenting a worse prognosis; similarly, adult patients are also considered to carry lower survival rates than infants (6,10).

In conclusion, RMS is an aggressive malignant soft tissue neoplasm that usually affects the head and neck region, including the oral cavity. Recent improvements in the therapeutic approaches significantly increased survival rates, but an early diagnosis is mandatory to achieve the appropriate management of these patients.
